# The Emerging Application of Itaconate: Promising Molecular Targets and Therapeutic Opportunities

**DOI:** 10.3389/fchem.2021.669308

**Published:** 2021-05-12

**Authors:** Jiaqi Lin, Jinxuan Ren, Dave Schwinn Gao, Yi Dai, Lina Yu

**Affiliations:** Department of Anesthesiology, The Second Affiliated Hospital, Zhejiang University School of Medicine, Zhejiang University, Hangzhou, China

**Keywords:** itaconate, itaconate derivative, inflammation, immunometabolism, therapy

## Abstract

Metabolites have recently been found to be involved in significant biological regulation and changes. Itaconate, an important intermediate metabolite isolated from the tricarboxylic acid cycle, is derived from cis-aconitate decarboxylation mediated by immune response gene 1 in mitochondrial matrix. Itaconate has emerged as a key autocrine regulatory component involved in the development and progression of inflammation and immunity. It could directly modify cysteine sites on functional substrate proteins which related to inflammasome, signal transduction, transcription, and cell death. Itaconate can be a connector among immunity, metabolism, and inflammation, which is of great significance for further understanding the mechanism of cellular immune metabolism. And it could be the potential choice for the treatment of inflammation and immune-related diseases. This study is a systematic review of the potential mechanisms of metabolite associated with different pathology conditions. We briefly summarize the structural characteristics and classical pathways of itaconate and its derivatives, with special emphasis on its promising role in future clinical application, in order to provide theoretical basis for future research and treatment intervention.

## Introduction

In the past decade, how intracellular metabolic changes control immunity and inflammation rose a resurgence of interest in immune metabolism (Kabat and Pearce, 2017). Tricarboxylic acid (TCA) cycle situates at the core of cellular metabolism and is the most common metabolic pathway in aerobic organisms (Martínez-Reyes and Chandel, 2020). TCA cycle is indispensable among many metabolic processes in activated macrophages. It can regulate the metabolic adaptability of macrophages and affect their effect. Itaconate is an important intermediate metabolite isolated from the TCA cycle, which has recently been found to participate in significant biological regulation and changes (Murphy and O'Neill, 2018).

Itaconate was first synthesized by chemical method in 1836 (Baup, 1836), and then it was confirmed that itaconate was synthesized by decarboxylation of cis-aconitate (Turner et al., 1840) and in 1955 immune response gene 1(Irg 1) was found to play an important role in mediating itaconate production in mitochondrial matrix (Lee et al., 1995). In fact, less was known about the molecular regulatory mechanisms behind the biosynthesis of itaconate led to its mainly application in industrial polymer synthesis since the 1950s (Rao et al., 2006). However, with the rapid development of metabolomics, an increasing number of potential mechanisms of itaconate has attracted more and more attention. The anti-inflammatory and anti-immune mechanisms of itaconate were described in two 2016 Nature articles (Bambouskova et al., 2018; Mills et al., 2018). In the process of inflammatory activation of macrophages infected by pathogens or stimulated by lipopolysaccharide(LPS), the intermediate products of TCA, such as succinate, citrate, and itaconate continuously accumulated to affect the expression of inflammatory factors and the concentration could reach the millimole level (Murphy and O'Neill, 2018). Itaconate may be the best example role as a dual viability of immunomodulators on both the transcriptional and metabolic levels and has been shown to participate in variety of the initiation and maintenance of the anti-inflammatory response in macrophages (Yu et al., 2019).

Since itaconate was pushed to the limelight as a key determinant and participated in macrophage stimulation as an important regulatory metabolite. Subsequently, a large amount of researches report that itaconate is a central and determinant component links three fields of immune, metabolism and inflammation together which is of great significance for further understanding mechanism of cellular immune metabolism and drugs development for the treatment of inflammatory and immune-related diseases in the future (Hooftman and O'Neill, 2019; O'Neill and Artyomov, 2019). This article reviews the biosyhthesis, structure, metabolic characteristics, classical pathway of itaconate, and summarizes its potential role on current clinical application in order to provide a theoretical basis for future research and treatment intervention.

## The Biosynthesis and Metabolism of Itaconate

**Figure 1:**
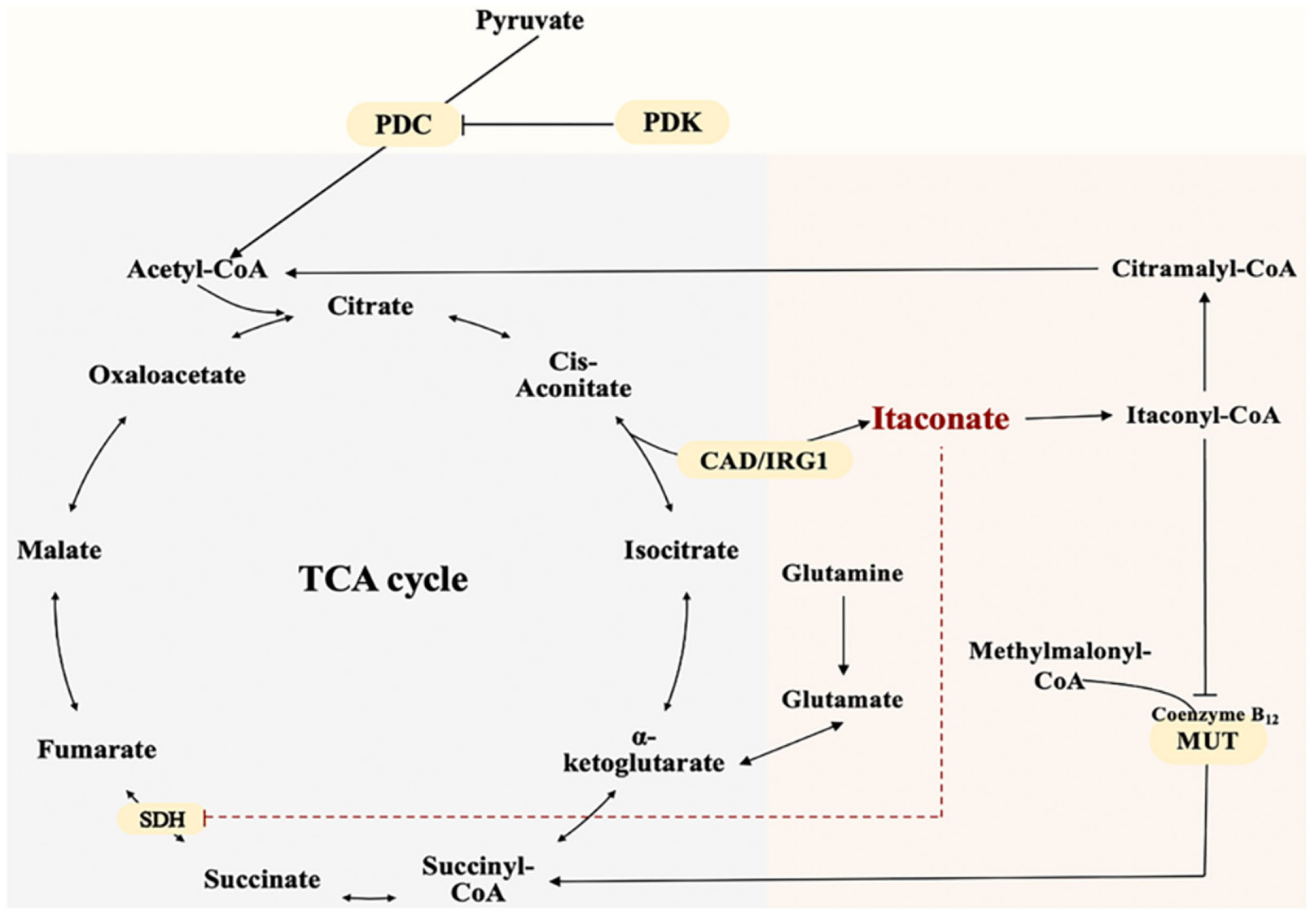
The Biosynthesis and Metabolism of Itaconate. Itaconate is produced by the decarboxylation of cis-aconitate encoded by aconitate decarboxylase 1. Itaconate inhibits SDH and accumulates Succinate. Pyruvate dehydrogenase complex catalyzes pyruvate into citrate precursor—acetyl-CoA.Itaconate is metabolized into itaconyl-coenzyme A. Itaconyl-coenzyme A inactivates mitochondrial CoA B12 thus inhibits methylmalonyl-CoA mutase and methylmalonyl-CoA conversion.

TCA cycle is a complex biological process involving a series of enzyme-catalyzed reactions for Adenosine triphosphate (ATP) production in the mitochondrial matrix (Martínez-Reyes and Chandel, 2020). The unique and crucial step in the biosynthesis pathway of itaconate is the decarboxylation of cis-aconitate produced by the dehydration of citrate. The decarboxylation is encoded by aconitate decarboxylase 1 (ACOD1), also named Irg1 (Michelucci et al., 2013). Irg1 overexpression leads to increased synthesis of endogenous itaconate (Wu et al., 2020). Catalytic conversion of isocitrate to α-ketoglutarate dehydrogenase further causes accumulation of citrate (itaconate precursor) (Nonnenmacher and Hiller, 2018). In addition, pyruvate dehydrogenase complex (PDC) catalyzes and controls the irreversible conversion of pyruvate into citrate precursor - acetyl-CoA (Michelucci et al., 2013). Phosphorylation of PDC by pyruvate dehydrogenase kinase 1 (PDK1)inactivates the enzyme and reverses this conversion (Denko, 2008; Meiser et al., 2016). Itaconate can be metabolized into itaconyl-coenzyme A (CoA), which inactivates mitochondrial CoA B12, resulting in a decrease in the activity of methylmalonyl-CoA mutase (MUT) and production of MUT-dependent branched-chain amino acids(BCAAs) (Zhu et al., 2020; Cordes and Metallo, 2021). Itaconate and its metabolite can affect other energy metabolism pathways besides the TCA cycle. The biosynthesis and metabolism of itaconate were shown as Figure 1.

## General Features of Itaconate

The lack mechanistic details of itaconate biology and regulatory function has led to the synthesis of several cell-permeable derivatives of itaconate, such as dimethyl itaconate (DI),4-octyl itaconate (4-OI) and ethyl itaconate (4-EI) to imitate the action characteristics of endogenous Itaconate. Thus, this section sets out to perform an introduction of the metabolic, electrophilic, and immunological properties of the itaconate and its three major derivatives reported in the literature to date: Itaconate, DI, 4OI, and 4-EI (Table 1).

**Table 1:** The chemical structures of itaconate and its derivatives.

		**Itaconate**	**DI**	**4-OI**	**4-EI**
		** 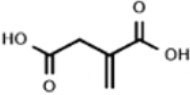 **	** 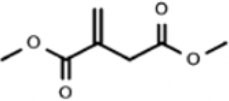 **	** 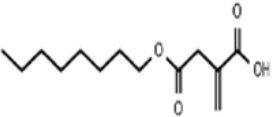 **	** 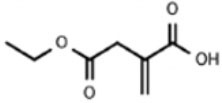 **
Chemical formular		C_5_H_6_O_4_	C_7_H_10_O_4_	C_13_H_22_O_4_	C_7_H_10_O_4_
Synthesize year		1836	1906	2018	1985
Molecular weight (g mol^−1^)		130.099	158.15	242.31	158.15
Concentration		7.5 mM	0.25 mM	0.25 mM	10 mM
Electrophilicity		±	+ +	+ +	±
Intracellular levels of itaconate		↑	–	–	↑
Immunological properties	Succinate	↑	–	–	–
	I-κBζ inhibition	–	+	+	–
	Pro-IL-1β	–	↓↓	↓	–
	Mature IL-1β	↓	↓	↓	↓
	IFN-β	↓	↓↓	↓↓	↓

*The chemical structures, molecular weight, electrophilicity and properties for itaconate and its derivatives a are summarized in this table*.

### Itaconate

Itaconate is an α,β-unsaturated dicarboxylic acid (C5H6O4) containing a double bond and two carboxyl groups (Robert and Friebel, 2016). It is an electrophile which has active chemical properties through forming a bond with a nucleophile by accepting an electron pair and to some extent showed similar characteristics to underlying enzymatic mechanisms (Robert and Friebel, 2016). Because of the conjugated unsaturated double bond structure of itaconate, it can affect the activity and function of substrate proteins by covalently modification of the cysteine residues in proteins in the way of Michael addition reaction, thereby exerting a strong potential in inhibitory effects on the inflammatory signaling pathway (Bambouskova et al., 2018; Swain et al., 2020). However, the high polarity and low electrophilicity of unmodified itaconate lead to its weak cell-permeability to satisfy further research. Previous work on itaconate are based on its derivatives. To better understand the real characterize of itaconate, Wang et al. designed a biorthogonal probe -Itaconate-alkyne (ITalk) in 2020 which retain the α, β-unsaturated carboxylic acid group and the long carbon chain (Qin et al., 2020). It was designed to directly capture modified proteins in living cells to identify bona fide targets of itaconate on a large scale. One thousand one hundred thirty-one itaconate-modified cysteine sites belonging to 1,926 itaconate-modified proteins including 199 hypersensitive targets were identified, including the previously known functional substrate proteins Aldolase A (ALDOA) and Kelch like ECH-associated protein 1 (Keap1) (Qin et al., 2019, 2020). Many key proteins heavily modified such as inflammasome, signal transduction, transcription, and cell death- related proteins involved in inflammatory immune response and host defense-related regulatory pathways suggesting that itaconate could affect macrophage function by regulating multiple pathways. This work greatly expanded a valuable resource database of biological roles of itaconate for further investigation.

### Dimethyl Itaconate

Itaconate derivative dimethyl itaconate (DI) first employed as a chemical experimental material is considered as “powered-up” version of itaconate (RajanBabu et al., 1999; Schmidt et al., 2008). The membrane-permeable ability of DI is attributed to a carboxyl group esterification on the 1-position (Lampropoulou et al., 2016). The conjugation effect of ester group prevents electron transfer and reduces the negative charge of itaconate and render its high acceptor reactivity in Michael addition. This is also the reflection DI significantly and acutely deplete glutathione (GSH) levels. The structural characteristics of DI makes it as a thiol-reactive metabolite to induce nuclear transcription factor erythroid-2-related factor 2 (Nrf2) and several Nrf2 target genes in a similar manner to Dimethyl fumarate (DMF), another potent Nrf2 activator (Carlstrom et al., 2019). The ability of DI to block LPS-induced IκBζ expression is the convincing proof of its strong electrophilicity (Bambouskova et al., 2018). However, in recent studies showed that short-term effect because of rapidly degradation or further metabolization of DI was not enough to release endogenous itaconate, hence is unlikely to be converted to intracellular itaconate (ElAzzouny et al., 2017). It could be inferred DI could not mimic itaconate and the broad metabolic effects by DI was not caused by intracellular itaconate accumulation but electrophilicity and covalent modification of metabolic enzymes (Swain et al., 2020). However, under the stimulation of LPS, treated with increasing concentrations of exogenous DI can increase the biosynthesis of itaconate (ElAzzouny et al., 2017).

### 4-Octyl Itaconate

Esterification on the 1-position of DI is a direct effect on the rapidly intracellular thiols reaction of Nrf2.And to overcome the limitations of DI, Mills designed a new itaconate surrogate, 4-octyl itaconate (4-OI) (Mills et al., 2018). The difference between 4-OI and DI is the location of octyl ester. Octyl ester group of 4-OI is located distal to the alkene than DI which makes 4-OI lower thiol reactivity similar to itaconate and more suitable to probe the physiological function of itaconate (Mills et al., 2018; Sano et al., 2020). The structural differences also made 4-OI lacks the electrophilic property which makes it a better candidate of Itaconate (Ryan et al., 2019). 4-OI can increase the level of itaconate by the way of hydrolyzation with or without the stimulation of LPS (Swain et al., 2020). This is the significant difference between DI and 4-OI, as previous discussion, DI only increase the biosynthesis of itaconate but cannot convert into intracellular itaconate (ElAzzouny et al., 2017). The main reason is the long carbon chain of 4-OI compared to DI to resistant to esterase hydrolysis. However,4-OI is better to mimic endogenous itaconate but not perfect enough. There are still difference such as hydrophobicity between itaconate and 4-OI on spectrum of proteins modify (Mills et al., 2018). All of these data suggest that the derivatives of itaconate mediate diverse pathways to protect against macrophage inflammation. Further studies should be explored for the extra side functions of DI and 4-OI.

### 4-Ethyl Itaconate

At present, there are few researches on 4-ethyl itaconate (4-EI), in an article comparing the metabolic, electrophilic, and immunologic profiles of itaconate and its derivatives has mentioned 4-ethyl itaconate (4-EI) (Swain et al., 2020). Esterified carbonyl group enhances the negatively charged, accordingly, this is why DI could induce a strong electrophilic stress response than that of 4-OI and endogenous itaconate, and makes DI play a better role of cell permeability (Mills et al., 2018; Swain et al., 2020). 4-EI has similar structure to DI, the electrophilicity of 4-EI is lower but polarity is higher than that of DI because only 4-carboxyl of 4-EI is esterified. It is also reflected in that 4-EI has no inhibitory effect on IκBζ. However, when given butylthionine sulfoxide (BSO), a non-electrophilic inhibitor of GSH synthesis, 4-EI has a consistent effect with DI on transcription factor inhibitor of NF-κBζ (IκBζ) (Bambouskova et al., 2018). It probably because the existence of GSH lead to a substantial decrease in 4-EI or DI reactivity. Another ethyl itaconate −1-ethyl itaconate(1EI) which the first position carboxyl group is esterified also make the inhibitory effects on IκBζ. Structural differences of different ester groups at different positions also correlate with functional differences (Bambouskova et al., 2018).

The study of itaconate as therapeutic molecules has generated excellent prospects in the pharmaceutical industry due to its low toxicity and high biological activity. None of the above three derivatives can well-simulate the ibona fide targets of itaconate, so there is an urgent need for a more perfect derivative to study the mechanism of itaconate more comprehensively.

## The Classical Mechanisms of Itaconate

**Figure 2:**
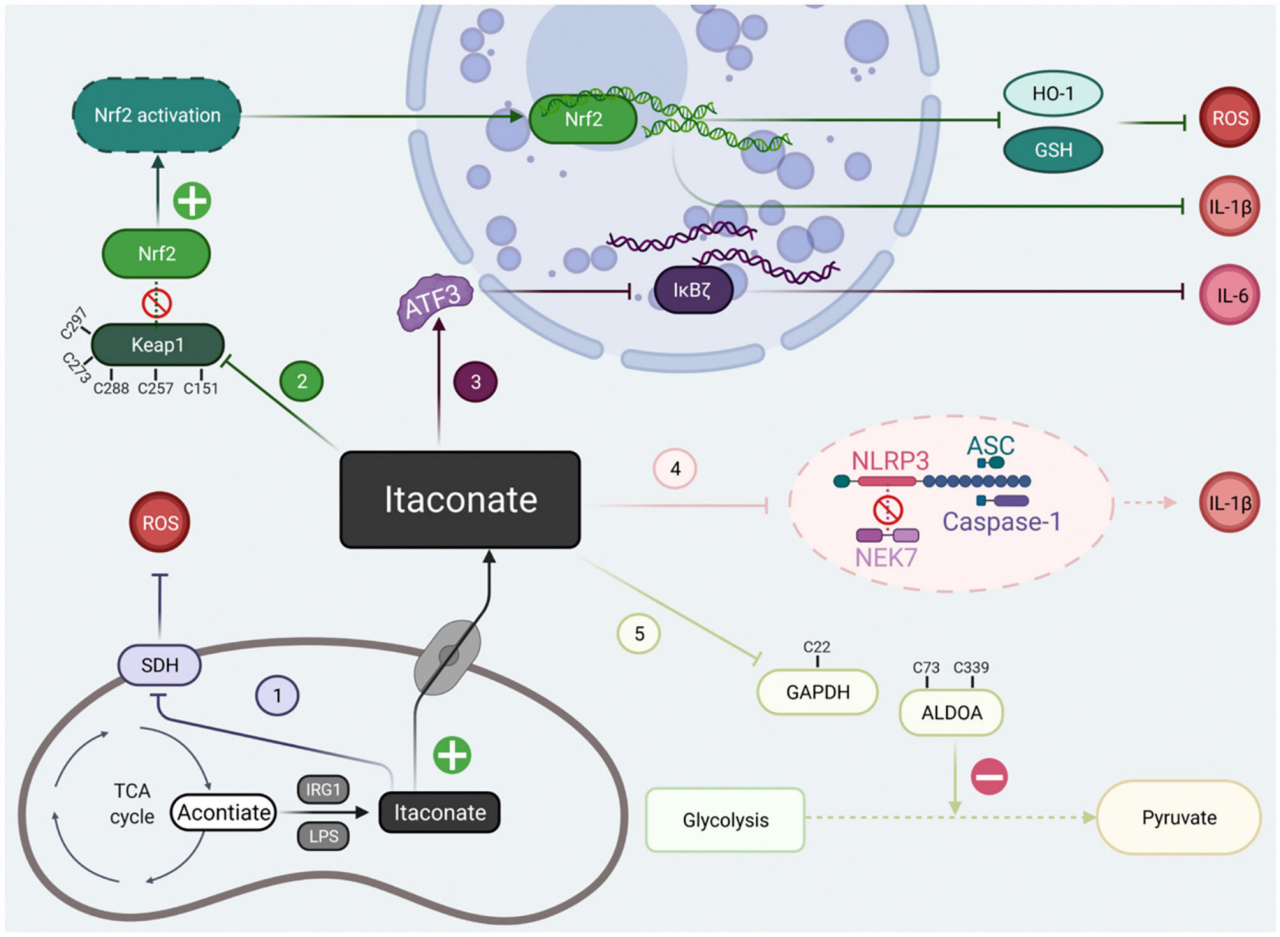
The classical signal pathways of itaconate that have been studied at present. The classical signal pathways of itaconate can be divided into five main types. (1) Itaconate mediated by IRG1 could inhibit due to structural similarity with succinate. (2) Itaconate covalently modify Keap1 cysteine 151 etc.to dissociate the combination of the Keap1-Nrf2, thus promote migration of Nrf2 to cell nuclei. (3) Itaconate increases the levels of ATF3 protein which translocated to the cell nuclei to inhibit IκBζ at the translational level. (4) Itaconate abolish NLRP3-NEK7 connection in a modification termed dicarboxypropylation on C548 of NLRP3 thus block NLRP3-dependent IL-1β release. (5) Itaconate inhibit glycolysis by alkylating cysteine 22 residues on GAPDH, cysteine 73, and 339 on ALDOA. Created with Biorender.

At present, there are two kinds of studies on itaconate: Irg1^−/−^ macrophages and the regulatory effect of itaconate derivatives. These two results were complementary and revealed that the regulatory mechanisms of itaconate involved alkylation on Keap1 to activate Nrf2, succinate dehydrogenase inhibition, activating transcription factor 3 (ATF3) induction to inhibit IκBζ activation, down-regulating glycolysis by GAPDH and ALDOA alkylation. The electrophilicity of itaconate and its derivatives are also indispensable in the process of metabolic regulation. Here, we will conclude the classical mechanism of itaconate to clarify its potential targets (Figure 2).

### Transcriptional Regulation

#### Irg1-Nrf2-Keap1

Nrf2 act as a multifunctional and indispensable player in modulating the inflammatory response and oxidative stress (Yang et al., 2014; Yamamoto et al., 2018). In the physiological homeostatic condition, unstimulated Kelch like ECH-associated protein 1 (Keap1) as a repressor combined with Nrf2 and sequestrated Nrf2 in the cytoplasmic (Cuadrado et al., 2019). Keap1 also facilitates Nrf2 degradation by ubiquitin-mediated proteasomal degradation pathway (Villeneuve et al., 2010). Mechanistically, Itaconate due to its electrophilicity structure of containing α, β-unsaturated carboxylic acid could make it form a modification of 2,3-dicarboxypropyl adduct to covalently modify cysteine 151 (C151) residues on KEAP1 by a Michael addition (Mills et al., 2018; Ryan et al., 2019). Alkylation of Keap1 cysteine residues disassociates the combination of Keap1-Nrf2 and liberates Nrf2 accumulated and translocated to the cell nuclei to initiate transcription of anti-inflammatory and anti-oxidant program. This opens the possibility that itaconate might extend the scope of the biological therapies by cysteine modification of numerous target proteins. Moreover, the effectiveness of Nrf2 regulator in response to LPS was chopped in Irg1- deficient macrophages (Mills et al., 2018).

#### ATF3- IκBζ

The mechanism of inflammation activation is a complex and continuous multi-step process. Except for Nrf2-dependent transcriptional regulation, a unique anti-inflammatory action of itaconate targets on ATF3-IκBζ pathway in a Nrf2-independent manner to mediate the inflammatory response (Bambouskova et al., 2018). IκBζ is a nuclear protein encoded by Nfkbiz gene that could control the release of proinflammatory cytokines interleukin-6 (IL-6), a product of the secondary but not primary transcriptional program responses to LPS on macrophages (Ghosh and Hayden, 2008). ATF3 is negative regulator of IκBζ, briefly, itaconate was posited to increase the levels of ATF3 protein which subsequent inhibit the expression of eukaryotic initiation factor 2 (eIF2a) so that inhibit IκBζ at the translational level (Bambouskova et al., 2018). Itaconate provides an additional link between metabolic perturbations and inflammatory signaling by targeting ATF3- IκBζ pathway.

### Metabolic Regulation

#### TCA Cycle Inhibition

Succinate dehydrogenase(SDH) also called mitochondrial complex II (CII) is an essential component for TCA cycle and cellular respiration via the electron transport chain (Mills et al., 2016; Bezawork-Geleta et al., 2017). SDH can oxidize and converts succinate to fumarate and eventually malate. The oxidation of succinate ultimately generates superoxide anion–reactive oxygen species (ROS), one of the most important targets of inflammation and oxidative stress (Tannahill et al., 2013; Huang et al., 2019). Itaconate was first demonstrated to inhibit SDH in 1949 (Ackermann and Potter, 1949) and further study showed succinate accumulation caused by itaconate put pro-inflammatory activated macrophages into a holding pattern (Cordes et al., 2016). Work by Lampropoulou et al. revealed that itaconate could directly block the enzyme activity of SDH using the biochemical assays (Lampropoulou et al., 2016). The main reason of SDH active site blockage arised from itaconate structural similarity with succinate and classical antagonist of SDH —malonate. Cordes et al. found that SDH inhibition by itaconate was reversible and occurred within seconds. SDH might be an early target of itaconate to affect metabolism and cell function rapidly (Cordes and Metallo, 2021). Overall, the anti-inflammation effect of itaconate by targeting on SDH strongly supports a prominent regulatory link between metabolism regulation and inflammation.

#### Glycolysis Inhibition

LPS stimulation changed the immunophenotype of macrophages to pro-inflammatory M1 and up-regulated glycolysis. Macrophages in the state of inflammation activation need to respond rapidly to stimulation by absorbing large amounts of glucose to produce abnormal bioenergy activity through glycolysis (Russell et al., 2019). Glyceraldehyde-3-phosphate dehydrogenase (GAPDH) as a rate-limiting enzyme in the sixth step of aerobic glycolysis regulates the total rate of the whole metabolic pathway. According to previous research, Endogenous fumarate with the similarity electrophilic α, β-unsaturated structural as itaconate could succinate the GAPDH and inhibit the enzymatic activity of GAPDH and thus inhibit inflammation progression (Blatnik et al., 2008). On the base of this similarity, further work by Liao et al. (2019) found the direct evidence that 4-OI directly alkylate cysteine 22 (C22) residues on GAPDH by similar dicarboxypropylation like Keap1 alkylation using the detection of U13C glucose tracing. The glycolytic blockage of 4-OI on GAPDH reduced the extracellular acidification rate and increased the intracellular oxygen consumption thereby activated the anti-inflammatory program and alleviated inflammation. Another research with the application of specific cysteine labeled probe have identified 260 itaconate-modified cysteines and found that itaconate could inhibit glycolysis by a negative feedback regulation on another two key enzymes to resist inflammation (Qin et al., 2019). Alkylation of two cysteines (Cys73 and Cys339) of ALDOA by itaconate have the same effect on the inhibition to impair glucose catabolism. However, alkylation of Cys84 of lactate dehydrogenase (LDHA) have more effect on lactate production than glucose accumulation. It probably because ALDOA controls the first step of glycolysis catalyzed reaction whereas LDHA convers pyruvate to lactate at the last step (Qin et al., 2019, 2020). This mechanism offers a novel insight of intracellular metabolites in the complex regulation function of inflammation progression.

## The Candidate Mechanisms of Itaconate

**Figure 3:**
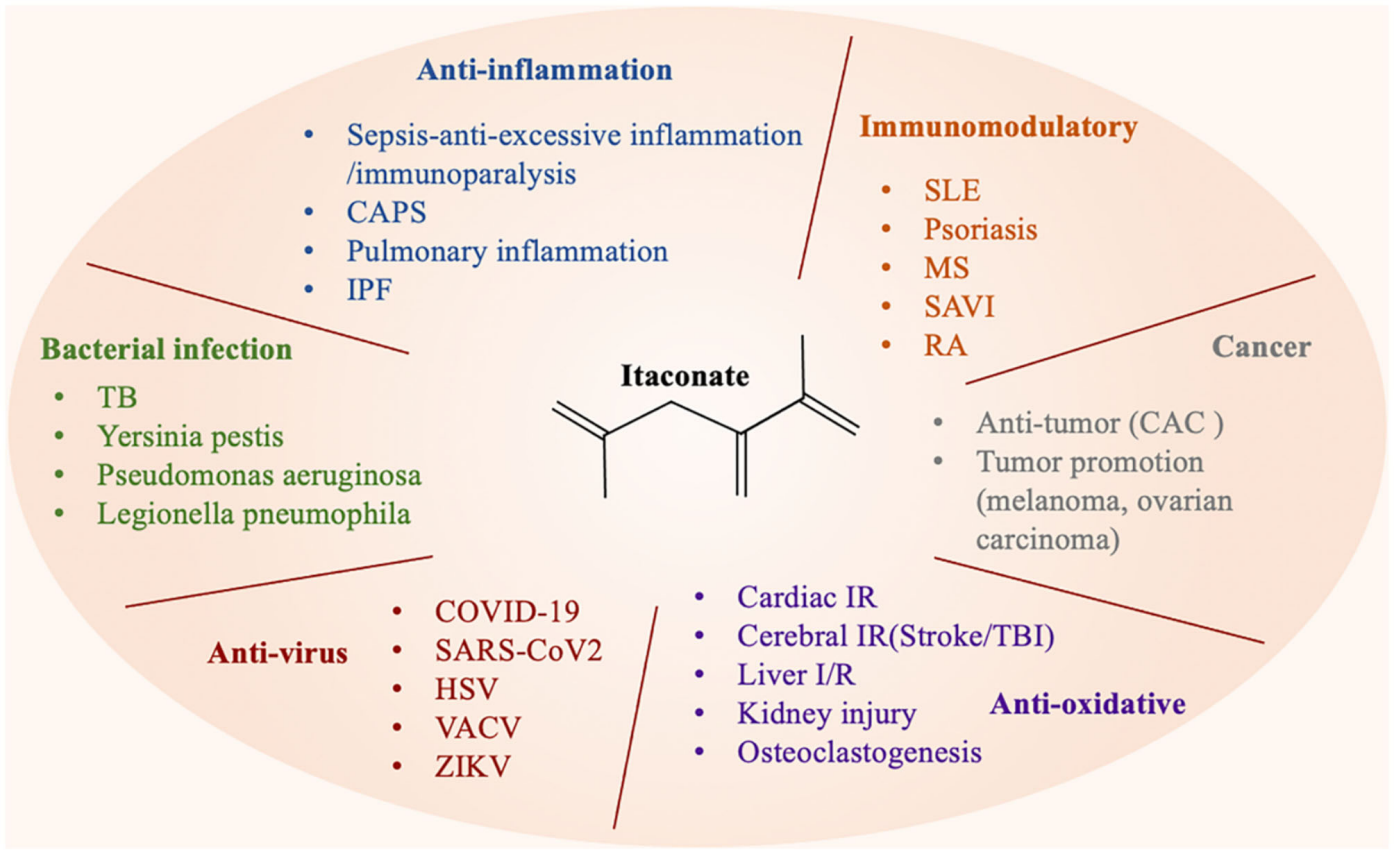
Itaconate can be involved in various types of diseases through a variety of regulatory ways.

Multiple studies have reported that the therapeutic effect of itaconate involved in many diseases, which can be described from the following aspects, including anti-inflammatory, immunomodulatory, antioxidant stress, anti-bacterial, and anti-virus (Figure 3). The application of itaconate in these diseases reflects its extensive regulatory potential (Tables 2, 3).

**Table 2:** The participation mechanisms of itaconate in different diseases.

**Diseases**		**Animals and cell**	**Agents and methods**	**Main mechanisms**	**References**
Anti-inflammation	Sepsis	C57(B6) mice BMDMs(mouse) PBMCs(human) RAW264.7 macrophages	LPS (Sigma; 2.5 mg/kg;100 ng/ml) OI (Sigma, 50 mg/kg, 125 μM)	Keap1/Nrf2-IFN	Mills et al., 2018
		BMDMs(mouse) C57(B/6J)mice	LPS (Sigma, 0.1 μg/mouse; 100 ng/mL) D-galactosamine (0.5 mg/g,0.5 mg/g) 4-OI (50 mg/kg; 200 μM)	Nrf2- HO-1/NQO-1	Zhang et al., 2021
		Whole blood (sepsis patients) C57(B/6J) mice Primary peritoneal macrophages RAW264.7 macrophages THP-1 cells(human)	LPS (Sigma,100 ng/mL; 1 μg/ml)	Itaconate induce immunoparalysis β-glucan reverse immunoparalysis made by itaconate	Li et al., 2013; Dominguez-Andres et al., 2019; Mainali et al., 2021
	CAPS	C57(B/6J) mice PBMCs, monocytes human HEK293T Cells	LPS (200 ng/mL) 4-OI (50 mg/kg)	NLRP3- IL-1β	Hooftman et al., 2020
	PM-Pulmonary inflammation	C57(B/6N,6J)mice BMDMs(mouse)	LPS (Santa;100 ng/ml) Itaconate (sigma;10 mM) 4-OI (sigma;0.25 mM)	ACOD1-SDH inhibition	Sun et al., 2020
	IPF	C57(B6)mice primary AMs, HLFs(human) HBEs(human)	Itaconate (Sigma, 0.25 mg/kg)	ACOD1-antifibrotic	Ogger et al., 2020
Immunomodulatory	SLE	THP-1 macrophages(human) PBMCs(human)	LPS (Sigma; 500 ng/mL) 4-OI (25 μM, 2 h)	Keap1-Nrf2-NF-κB	Tang et al., 2018
	Psoriasis	BMDMs(mouse) C57(B6,B/6N,6J) mice BV2 microglial cell	LPS (Sigma; 100 ng/mL) DI (Sigma; 20 mg/mouse, 250 μM) DMF (Sigma, 50 μM)	DI-IκBζ- IL-17	Bambouskova et al., 2018
	Multiple sclerosis	C57(B6),SJL/J mice Microglia(mice) Mononuclear cell(mice)	LPS (Sigma; 100 ng/mL) DMI (Sigma;400 mg/kg, 150 μM)	MMP3, MMP9 inhibition inhibite Th1/Th17 differentiation and infiltration to CNS	Kuo et al., 2020
	SAVI	THP-1 cells, PBMCs,HaCat HEK293T, A549 cells(human)	4-OI (Aarhus University;125 μM, 200 μM)	Nrf2-STING-IFN	Olagnier et al., 2018
Anti-oxidation	Heart	C57(B6, B/6N) mice BMDM (mouse) RAW264.7 macrophages	LPS (Sigma; 100 ng/mL) DI (4 mg/kg/min;0.25 mM)	SDH inhibition HIF-1α/IL-1β, IL-18 reduction not TNF-α production	Lampropoulou et al., 2016
	Brain	C57(B/6J) mice Primary cortical neurons, astrocytes(SD rat)	Itaconate (15 mg/kg/min)	SDH inhibition ROS/RNS reduction	Cordes et al., 2020
		C57(B6) mice	DMI (Sigma, 20 mg)	Inhibited toxic conversion of microglia	Zhang et al., 2019
	Liver	C57 (B/6N, B/6J) mice hepatocytes(human,mouse) NPCs(mouse)	4-OI (25 mg/kg, 62.5/125 μM)	IRG1-Nrf2- antioxidant	RajanBabu et al., 1999
	Kidney	SD Rat HK-2 cell	4-OI (1, 10 mg/kg; 1, 10, 30,1 00 μmol/L)	4-OI-TGF-β/Smad- NF-κB	Tang et al., 2019
	Bone	C57(B6)mice BMMs(mouse) OB-6 (human)	LPS (Sigma;10 ng/ml) DI (Sigma,10 μM) 4-OI (MedChemExpress, 50 mg/kg)	Nrf2—Hrd1- ubiquitination pathway	Sun et al., 2019; Zheng et al., 2020
Cancer	CAC	C57(B6)mice	DI (10 mg/kg)	Inhibited IL-1β/CCL2 and MDSC Infiltration reduced CAC risk	Wang et al., 2020
	Peritoneal tumors	C57(B6)mice Peritoneal Mφ(mouse) B16 melanoma, 3LL, and MC38 PBL(human)	/	Irg1-ROS-MAPK(promote cancer)	Weiss et al., 2018
Anti-bacterial	Tuberculosis Yersinia pestis Pseudomonas aeruginosa et al.	C57(B6,B/6N)mice SJL (CD45.1) mice	Itaconate (Sigma, 0.25 mM)	Irg1/NF-κB B12-dependent MCM inhibition ICL inhibition	Nair et al., 2018; Ruetz et al., 2019; Wang et al., 2019
Antivirus	COVID-19 SARS-CoV2 HSV-1 HSV-2 VACV ZIKV	C57 (B/6N, B/6J) mouse Primary cell (human, mouse) PBMCs(human)	4-OI (125 μM, 150 μM)	IRG1- RIPK3 SDH inhibition NRF2- IRF3	Dalglish, 2020; Olagnier et al., 2020; Song J. W. et al., 2020; Zhao et al., 2020

*This table summarizes literary papers focusing on the experimental evidence from animal studies (i.e., experimental model and methods, agent dosage, and mechanisms)*.

**Table 3:**
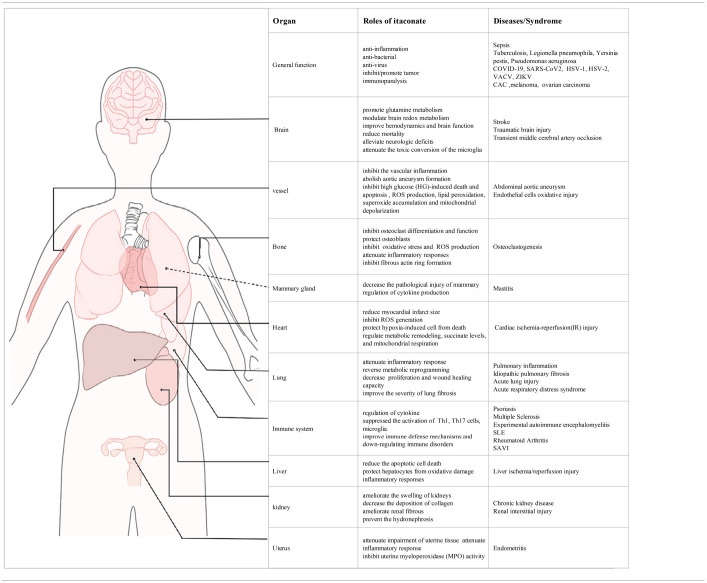
The role of itaconate and its potential clinical application.

*Itaconate plays multiple roles in different tissues and disease conditions and this table highlights to show the utility of itaconate for potential clinical*.

### The Effect of Itaconate in Anti-inflammation

#### Sepsis

The definition of Sepsis 3.0 is the life-threatening organ dysfunction caused by a host's inappropriate response to infection, emphasizing the imbalance between inflammation and immune homeostasis in sepsis progression (Cecconi et al., 2018). During the early stage of infection, the monocyte-macrophage system and neutrophils are continuously activated under the stimulation of pathogens, resulting in a massive release of pro-inflammatory cytokines (van der Poll et al., 2017). In an LPS-induced sepsis mice model, administration of DI or 4-OI protected mice from death and inhibited excessive inflammation. Zhang et al. showed that the administration of DI enhanced survival rate, decreased serum level of tumor necrosis factor-α (TNF-α) and IL-6, and ameliorated lung injury in septic mice and BMDMs via promoting the expression of Nrf2 and its downstream factor Heme Oxygenase 1(HO-1) and quinoneoxidore-ductase 1(NQO-1) (Zhang et al., 2021), which was consistent with that of Mills, Evanna L (Mills et al., 2018). However, as the disease develops, the high inflammatory storm evolves into immunosuppression or immunoparalysis, which increases the susceptibility to secondary infections (Biswas and Lopez-Collazo, 2009). Irg1 was highly upregulated both in sepsis patients and mice with LPS tolerance models, which indicated that itaconate might be involved in the immunoparalysis of sepsis (Li et al., 2013). However, the continual decarboxylation of the TCA intermediate cis-aconitate to itaconate resulted in a long-term, low-energy state, which may be a pathological state exacerbated immunoparalysis (Zhu et al., 2020). Zhu et al. found when reversed the increase of itaconate rejuvenated the anabolic TCA cycle, and increased the ATP produced by the mitochondrial electron transport chain thereby improved immune tolerance in septic shock model and THP-1 human monocytes during sepsis-like inflammatory responses (Zhu et al., 2020; Mainali et al., 2021). Intriguingly, monocytes treated with β-glucan can inhibit the expression of Irg1 induced by LPS and revert the potential harmful effects of itaconate (Dominguez-Andres et al., 2019). These studies have thoroughly and carefully distinguished the potential applications of itaconate in different periods of sepsis. The proper anti-inflammatory effect of itaconate during the excessive inflammation period the reduction of itaconate in the immunoparalysis phase can effectively control the progression of sepsis.

#### CAPS

Cryopyrin-associated periodic syndrome (CAPS) is an inherited autoinflammatory disease with hyperactive nod-like receptor protein 3 (NLRP3) (Ohnishi et al., 2012), an inflammasome as one of the best characterized intracellular multi-protein complexes is a critical determinant of a wide range of different autoinflammatory conditions (Swanson et al., 2019). The control of NLRP3 inflammasome activation may be a critical role in potential target of treatment (Prochnicki and Latz, 2017). A new research published on journal of Cell Metabolism found that 4-OI could be converted to intracellular itaconate by macrophage and the effect of endogenous itaconate was confirmed to specific targeting of NLRP3 (Hooftman et al., 2020). Itaconate could abolish NLRP3-NEK7 connection in a modification termed dicarboxypropylation on C548 of NLRP3. They also observed that 4-OI blocked NLRP3-dependent interleukin-1β (IL-1β) release from PBMCs isolated from CAPS patients, while SDH inhibitor did not have the same effect. Indeed, itaconate modulated LPS-regulated genes related to inflammasome activation and function, a significant portion of prototypical NLRP3-activating conditions. The targeting of NLRP3 by itaconate provides therapeutic potential for the treatment of NLRP3-driven disorders.

#### Pulmonary Inflammation

Macrophage-driven lung inflammation is associated with particulate matter (PM) air pollution (McGlade and Landrigan, 2019). Exposure to pollution particles led to the increase of mitochondrial oxygen consumption rate and ROS eventually induce the production of TNFa and IL-6 (Soberanes et al., 2019). RNA-Seq analysis showed that Acod1 gene encoding the enzyme producing itaconate was significant activated in the polluted environment exposed to pulmonary macrophages in a time-dependent manner. Itaconate was indispensable for regulating its downstream antioxidant genes and inflammatory genes (Sun et al., 2020). If treated with 4-OI, the Nrf2 pathway would be opened and succinate was also accumulated which eventually reduced pulmonary inflammation. Similar results could be obtained when treatment before exposure to contamination. Itaconate was also found to alleviate idiopathic pulmonary fibrosis (IPF) through changing macrophage phenotype and function (Wynn and Vannella, 2016; Allden et al., 2019). A recent study found that the expression of ACOD1/itaconate was obviously reduced in patients with IPF, this reduction was proved to exacerbate more disease severity of pulmonary fibrosis in mice (Ogger et al., 2020). Administration or inhalation of exogenous itaconate reversed the formulation of pulmonary fibrosis through changing the fibroblast metabolic phenotype, decreased proliferative capacity, and limited wound healing of human lung fibroblasts. 4-OI was also found play an extremely important role in alleviating acute lung injury (ALI) or acute respiratory distress syndrome (ARDS) by inhibiting the expression of the downstream inflammatory cytokines (IL-β, IL-6, and TNF-α) and ROS to increase the activity of antioxidants in lung tissue (Li et al., 2020).

#### Abdominal Aortic Aneurysm

Itaconate, kind of inflammation-related endogenous metabolites serve as a novel and inexpensive therapeutic target to control the progression of vascular inflammation disease -abdominal aortic aneurysm (AAA) (Song H. et al., 2020). And Irg1/itaconate pathway was involved in the formation of AAA. Exogenous itaconate addition in apolipoprotein E-deficient (Apoe^−/−^) mice suppressed the initiation and progression of AAA and downstream inflammatory protein while Irg1 deficiency reverted the effect of inhibitory. Overexpression of Keap1 or transferred Cys151S mutant Keap1 vector also abolished the activation of Nrf2 induced by itaconate.

#### Mastitis/Endometritis

In two studies explored about the protective effect of DI to prevent the pathology inflammation of mastitis/endometritis diseases (Zhao et al., 2019; Xu et al., 2020). Injection of DI markedly decreased the production of pro-inflammatory cytokines, and increased the expression of Nrf2, HO-1 in LPS-induced mastitis and endometritis mice. It also inhibited the signal pathway of TLR4 and phosphorylation of p65 nuclear factor kappa B (NF-κB). Their researches investigated that DI may serve as a potential candidate to protect against pathological damage of mastitis/endometritis.

### The Effect of Itaconate in Immunomodulatory

#### Psoriasis

Immunometabolism, as a burgeoning field has linked intracellular metabolic pathways to immune-mediated inflammation conditions (Diskin et al., 2020). Itaconate is perhaps the crucial determinant of immune-mediated diseases because of its multiple immunomodulatory and anti-inflammatory effects. Psoriasis is well-known as an autoimmune disease of the joints and skin with inappropriate T cell activation. The transcriptional activator IκBζ induced by IL-17A-treated epithelial cells plays a key role in mediating the orchestrates downstream inflammatory responses in immune-related conditions, psoriasis (Bambouskova et al., 2018; Bertelsen et al., 2020). DI pretreatment interfere with the production of IκBζ in a way of electrophilic stress mediated by ATF3, a key mediator of the Nrf2-independent way and downregulate IκBζ protein correlated genes in primary keratinocytes stimulated by IL-17A (Bambouskova et al., 2018). No significant changes of scaling or oedema of the skin was observed in a mouse model of psoriasis-like pathology condition treated with DI. And daily DI administration has a favorable safety profile with little effect in the heart and the liver. Targeting the DI–IκBζ regulatory axis may be a new approach to subside the symptoms of autoimmune condition.

#### Multiple Sclerosis

Multiple sclerosis (MS) is a progressive demyelinating destruction associated with immune-mediated pathogenesis of central nervous system (Faissner et al., 2019). A recent study showed the immunomodulatory effect of DI in an Experimental autoimmune encephalomyelitis (EAE) animal model commonly represented MS (Kuo et al., 2020). DI is capable of decreased production of MMP3 and MMP9 which lead to the lessen of BBB disruption (Brown, 2001; Mirowska-Guzel et al., 2009). DI suppressed the activation of microglia thus decreased the infiltration and differentiation of encephalitogenic Th1 and Th17 cells in EAE through a Nrf2-independent mechanism. The multiple cellular and molecular defense pathway of DI ameliorated the severity of chronic EAE condition. Dimethyl Fumarate (DMF) is the traditional immunomodulatory drug of MS and psoriasis dependent on GAPDH blockage and aerobic glycolysis inhibition (Kornberg et al., 2018). Itaconate has the same effect on GAPDH. However, inferred from the mechanism of DMF, whether there are other mechanisms of itaconate in the treatment of MS need to be further confirmed.

#### SLE

Systemic lupus erythematosus (SLE) is a common condition characterized by the dysregulation of pro- and anti-inflammatory cytokines (Tsokos et al., 2016). Treatments targeted inflammatory cytokines which induces a significant clinical improvement in patients, such as BENLYSTA and other biological agents have been widely used (Lee et al., 2011). Tang et al. (2018) reported that TNF-α, IL-1β, and IL-6, were significantly decreased in the PBMCs of SLE patients and THP-1 cells treated with OI. The probably mechanism they found was that OI disassociated Keap1 from Nrf2 and exerted a potent anti-inflammatory effect by the accumulation, activation and nuclear translocation of Nrf2 protein.

#### Rheumatoid Arthritis

Rheumatoid arthritis (RA) is a debilitating immune-mediated disease of global prevalence (Weyand and Goronzy, 2021). The variation of plasma metabolic spectrum in patients with rheumatoid arthritis is instructive when they begin routine anti-rheumatic drug (CDMARD) treatment (Daly et al., 2020). Plasma samples from an early RA randomized strategy study (NCT00920478) were analyzed by untargeted metabolomics analysis. A total of 9 metabolites were significantly correlated with the decrease of rheumatic activity score after using CDMARD, the main components of which were itaconate and its derivatives of coenzyme A. After CDMARD (mainly methotrexate) treatment, the increase of itaconate was related to the improvement of rheumatic activity score and the decrease of C-reactive protein (CRP) level. This is consistent with its anti-inflammatory effect, which suggests that itaconate may be a sign like CRP of improvement in patients. Another recent study described itaconate as a key marker in the progress of inflammatory arthritis in Tg197 mice model, and found that the level of itaconate was increased when TNF-α was blocked (Michopoulos et al., 2016). Further study of itaconate pathway may reveal new important insights into the regulation of immune function and the pathogenesis of rheumatoid arthritis. It may also reveal new clinical markers of disease activity and treatment response.

#### SAVI

Stimulator of interferon genes (STING)-associated vasculopathy(SAVI)caused by mutation of TMEM173 gene is a system disruption of inborn innate immune disorders characterized by neonatal onset of autoinflammation diseases (Ahn and Barber, 2014). STING as an adaptor protein could induce antiviral type I IFN signaling thus signals downstream of viral (Siedel et al., 2020; Donnelly et al., 2021). Nrf2 as an important negative regulator engage on STING repression and destabilization. 4-OI that linked to Nrf2 activation was sufficient to decrease 2-fold levels of TMEM173 mRNA and the STING related-mRNA in HEK293T cells. The treatment of itaconate also inhibited the release of STING-dependent type I IFNs from SAVI-associated fibroblasts condition. Itaconate may gain a newfound foothold and valid target in the rare STING-associated inflammatory disorders (Olagnier et al., 2018).

### The Effect of Itaconate in Anti-oxidative

#### Ischemia-Reperfusion (IR) Injury

Ischemia-reperfusion(I/R) injury is a complex pathological condition which drives an imbalance of injurious metabolic processes between oxidative stress and anti-oxidant defense systems (Chamorro et al., 2016). Itaconate as a significant portion of physiological regulatory mediators participates in upregulation of succinate levels, production of inflammatory cytokine, mitochondrial respiration and directionality of the electron transport chain. A recent study demonstrated that intravenous infusion of DI induced reduction in myocardial infarct size (Lampropoulou et al., 2016) by inhibiting SDH in cardiac I/R injury on dose-dependent (Kula-Alwar et al., 2019). Pretreatment with DI exerted anti-inflammatory effects by inhibiting the expression of inducible nitric oxide synthase (iNOS) protein and secretion of IL-12p70 and IL-6 whereas no significant change of TNF-α was found indicating that DI-administrated probably did not affect NF-κB-dependent gene expression. DI impaired IL-1β production induced by not only prototypical NLRP3- dependent inflammasome activation but also absent in melanoma-2 (AIM-2)-activating conditions revealing that itaconate could exert a broader regulatory effect.

Cordes et al. found that exogenous itaconate also suppressed SDH and dramatically affected the expression levels of Hmox1, Nqo1, and Gpx1 genes, initiating the transcription of multiple antioxidant and anti-inflammatory protein in cerebral I/R injury model (Cordes et al., 2020). Zhang et al. further showed that intraperitoneal administration DI significantly alleviated neurologic deficits and promoted neural functional recovery on day 3 after surgery (Zhang et al., 2019). In addition, DI was also showed to inhibit the conversion of M1 microglia which was a critical determinant of pro-inflammatory effects. 4-OI activated Keap1-Nrf2 signaling to protect neuronal cells from hydrogen peroxide (Liu et al., 2018).

Oxidative stress is also a major contributor to liver I/R injury apart from cardiac and brain Yi et al. (2020) found that IRG1-itaconate-Nrf2- antioxidant pathway protect hepatocytes from oxidative damage in liver ischemia–reperfusion and hypoxia/reoxygenation. They observed that Irg1 deficiency mouse rendered more susceptible to systemic and local inflammation and liver injury. Administration of 4-OI ameliorated oxidative stress and hepatocyte cell death in a manner of Nrf2-driven signaling.

#### Diabetes Mellitus-Induced Vascular Injury

Pathological change of vascular endothelial cell injury is the commonest cause of diabetes vasculopathy. Pre-treatment with OI was found have the protection in human umbilical vein endothelial cells (HUVECs) from high glucose (HG)-induced oxidative injury in mimicking Diabetes mellitus (Tang et al., 2019). The anti-HG protection was probably that OI abolished the association of Keap1-Nrf2 leading to the accumulation of Nrf2 translocated to cell nuclei. In consistent with other reports, this is also the reason of superior anti-oxidant activity in OI by inhibiting the production and accumulation of ROS or superoxide, decreasing the lipid peroxidation and mitochondrial depolarization. And OI nullified to against HG when Nrf2 was genetically silenced or depleted or cells transfected by Keap1 Cys151S mutation vector.

#### Renal Fibrosis

Another study has found the renoprotection made by itaconate on renal fibrosis which is recognized as an inevitable pathological progression of all chronic kidney disease (CKD) (Tian et al., 2020). After OI treatment, creatinine (CRE) and blood urea nitrogen (BUN) were reduced thus prevented the occurrence of hydronephrosis. OI ameliorated renal perivascular fibrous tissue hyperplasia by the suppression the expression of transforming growth factor-β(TGF-β)/Smad and NF-κB pathways. These results also indicate the clinical application of OI through the antioxidant stress potential.

#### Osteoclastogenesis

4-OI affected the suppression of oxidative injury induced by hydrogen peroxide (H_2_O_2_) in osteoclast-related diseases. Sun et al. found that the concentration of itaconate was lower in estrogen-deficient mice analyzed by LC-MS assay and the deficiency of Nrf2 was found to induce osteoclastogenesis (Sun et al., 2019). 4-OI could inhibit bone resorption, osteoclast differentiation and attenuated bone loss *in vivo* and *vitro*. The underlying mechanism was exogenous 4-OI modulated the ubiquitination-mediated degradation of Nrf2 thereby enhanced Nrf2 expression by suppressing the association between Nrf2 and E3 ubiquitin ligase (Hrd1) in a time-dependent manner. Furthermore, they also found that DI did not have the same effect on osteoclast function indicate that general electrophilic stress was probably not sufficient to induce the inhibition of osteoclastogenesis. In another research Zheng et al. (2020) found 4-OI protected OB-6 cells and primary murine osteoblasts from oxidative injury, cell death, and apoptosis treated by H_2_O_2_ through disassociation of Keap1 and Nrf2.

### The Effects of Itaconate in Cancer

Recent studies have also found that itaconate plays a vital role in cancer immunometabolism. The anti-cancer property of itaconate has been reported in colitis-associated colorectal cancer (CAC) (Wang et al., 2020). Administration of DI inhibited the secretion of cytokines IL-1β and chemokine ligand 2 (CCL2) in intestinal epithelial cells, reduced the infiltration of inflammatory macrophages to the tumor microenvironment (TME), thus alleviated the high inflammatory state of ulcerative colitis. The effect of itaconate was also accompanied by suppressing the cytotoxic T cell differentiation and their infiltration of myeloid-derived suppressor cells (MDSCs), thereby reducing the risk of CAC development. In other studies, itaconate level was proved to correlate with cancer progression and the unfavorable prognosis in certain tumor types (Weiss et al., 2018). As the most crucial component in TME, tumor-associated macrophages (TAMs) can secrete various mediators such as cytokines (IL-10) and growth factors that promote tumor growth, invasion, and metastasis (Noy and Pollard, 2014; Vitale et al., 2019). The peritoneal tissue-resident macrophages(pResMφ) were found to upregulate Irg1 expression and increase itaconate level in B16 melanoma and ID8 ovarian carcinoma (Weiss et al., 2018). Itaconate was shown to increase oxidative phosphorylation (OXPHOS) with resultant production of ROS, which mediated mitogen-activated protein kinases (MAPK) activation to promote tumor progression. Conversely, when the conversion to itaconate was inhibited in Irg1^−/−^ mice, a significant tumor volume reduction was observed. Furthermore, itaconate promoted ROS in pResMφ through OXPHOS, which was mainly driven by the Nicotinamide adenine dinucleotide (NADH) cycle, a succinate dehydrogenase-independent mechanism (Basit et al., 2018). Monocytes isolated from the ascetic fluid of ovarian cancer patients expressed high levels of Irg1. Similarly, another article pointed out that Irg1 played an oncogene-like role in glioma, leading to poor prognosis (Pan et al., 2014). Further research is needed to fully identify the regulatory effects of itaconate on different types of tumors.

### The Effects of Itaconate in Bactericidal Action

Tuberculosis (TB) caused by Mycobacterium tuberculosis (Mtb) is the severe bacterial disease in terms of its high mortality and prevalence worldwide (Shin et al., 2011). In 2011, Shin et al. found that itaconate was increased only in Mtb-infected lung tissue for the first time by using metabolic profiles. However, the reason itaconate increased after an aerobic infection did not clarify. Nair et al. found a role for the IRG1-itaconate axis in Mtb-infected mice. Cell-intrinsic expression of Irg1 in alveolar macrophages and some dendritic cell subsets is dispensable for neutrophil recruitment and inflammation regulation. They next observed that itaconate was dramatically decreased in Irg1^−/−^mice. Analogously, addition of exogenous itaconate treatment reverses the induction of inflammatory by modulating chemoattractant genes downstream of NF-κB signaling in a (Irg1^−/−^) Mtb-infected mice. These results suggested that itaconate and Irg1 played a critical role as an endogenous antibacterial effector molecule on Mtb growth (Nair et al., 2018). Another study found that the bactericidal effects of itaconate was probably due to its inhibition of B12-dependent methyl malonyl-CoA mutase (MCM) which is needed for Mtb growth on propionate as the sole carbon source. what's more, itaconate can be acylated to itaconyl-CoA (I-CoA) via a C5-dicarboxylate pathway (Ruetz et al., 2019). I-CoA also derailed the activation and repairment of Mtb MCM and the radical suicide inactivation mechanism of I-CoA was forming a markedly air-stable biradical binding with MCM (Wang et al., 2019). Moreover, a study showed that itaconate modified the proteome of Legionella pneumophila containing vacuoles to restrict intracellular bacterial pathogens (Naujoks et al., 2016). In addition, itaconate was found to restrict bacterial growth in culture by inhibiting isocitrate lyase (ICL) (a key enzyme that supports bacterial growth during infection (McFadden and Purohit, 1977). Meanwhile, the inhibitory effect of itaconate was also found in bacteria that did not express ICL enzyme, which may be due to the inhibition of acetic acid assimilation by inhibiting propionyl-CoA carboxylase (Yang et al., 2020). Some bacteria such as Yersinia pestis and Pseudomonas aeruginosa which carried genes encoding enzymes of itaconate degradation can promote pathogenicity and survival (Rao and McFadden, 1965; Rittenhouse and McFadden, 1974; Chen et al., 2020; Riquelme et al., 2020). Activated macrophages have been shown to produce itaconate, suggesting that these immune cells may employ this metabolite as a weapon against invading bacteria. Itaconate can exhibit bactericidal effects under acidic conditions similar to the pH of a macrophage phagosome (Hersch and Navarre, 2020). These studies showed that itaconate has been proposed to have direct bacteriostatic activity (Williams and O'Neill, 2018). It also implied that there was a complex evolutionary relationship between itaconate and bacteria.

### The Effect of Itaconate in Antivirus

Mounting evidence discovered that an unexpected intersection between itaconate and immune activation is intricately linked with antivirus strategies. Zika virus (ZIKV), an emerging human pathogenic virus can cause significant neurologic injury by access the central nervous system (CNS) and has become an increasingly global public health challenge (Zhao et al., 2020). The production of itaconate as a direct downstream effector of ZBP1- and RIPK- IRG1 dependent transcriptional program could ameliorate viral pathogenesis in the CNS. The underlying mechanisms was probably that IRG1, a potential antiviral gene restricted replication and neuronal infection of ZIKV in neurons in a cell-intrinsic manner. Besides itaconate produced by IRG1 could alter neuronal metabolism by inhibiting the activation of SDH and a cellular environment is thereby to exert a global suppression on viral replication.

As every knows, the coronavirus disease 2019 (COVID-19) has rapidly posed an unprecedented global pandemic with high morbidity, mortality, social disruption, and economic instability. But there are limited options on the prevention and treatment of this global health emergency (Dalglish, 2020). Song J. W. et al. (2020) first found there were significant difference of metabolites related to COVID-19 patients investigated in logistic regression models. Compared serum lipidome and metabolome with different stages of COVID-19 patients and healthy crowds revealed that itaconate was declining progressively with the severity of COVID-19. Works by Olagnier et al. analyzed the differentially expressed genes transcriptome between lung biopsies from COVID-19 patients by publicly available transcriptome data at first (Olagnier et al., 2020). They discovered that the expression of antioxidant genes driven by Nrf2 were significantly suppressed in COVID-19 patients. 4-OI and DMF as Nrf2 inducers significantly decreased the release of progeny virus particles and the level of virus RNA in different cells infected with SARS-CoV-2. The same antiviral phenomenon treated by 4-OI occurred in other human pathogenic viruses except for vesicular stomatitis virus (VSV). 4-OI retained the antiviral replication capacity, whereas the antiviral mode of action probably not rely on the classical IFN way due to its interruption of interferon regulatory factor 3 (IRF3) activation and dimerization. These data pointed out that SARS-CoV2 targeted the Nrf2 antioxidant pathway and 4-OI as a Nrf2 inducer could perhaps be a rapidly applicable antivirus.

## Perspective and Conclusion

Since the key metabolic regulation of itaconate in macrophages was revealed, people have begun to recognize the complex interaction between metabolism, immunity, and inflammation, which provides us a new perspective for the treatment of immune inflammation-related diseases (Kabat and Pearce, 2017; Martínez-Reyes and Chandel, 2020). The signal pathways of itaconate have been summarized on Figure 2. However, in the research of Wang et al. we found that more targets of itaconate related to signal transduction, apoptosis and other signal pathways have not been discussed, and more studies are needed to supplement the mechanism of itaconate (Qin et al., 2020). Therapeutic value of itaconate in a variety of disease models (inflammation, immunomodulatory, antibacterial and antiviral, etc) can extend itaconate to the future clinical application. For example, itaconate has similar characteristics to other Nrf2 activators, while some Nrf2 agonists dimethyl fumarate have been proved to be effective in the treatment of some inflammatory diseases and used in the clinical treatment of multiple sclerosis (Kornberg et al., 2018; Carlstrom et al., 2019). This also suggests that itaconate is a very promising target for the treatment of diseases, but the current disease models are still not comprehensive enough. Further expansion of the types and models used in these studies will benefit the field of itaconate biology. Itaconate as an endogenous metabolite, will be a promising therapeutic in clinical treatment because of its low toxicity. However, most of the current therapeutic potential results come from animal models or *in vitro* studies. Moreover, we still need to consider that excessive immunosuppression will lead to immune paralysis and reduce the body's resistance to external infection. Although there are very few studies on itaconate in cancer, we cannot ignore its effect of tumors growth, and the progress of anti-tumors still needs more researches to support. The effect of itaconate on anti-inflammation is just like the discovery of the role of microbial metabolites as antibiotics in the 20th century may lead to the opening of the anti-inflammatory treasure house of metabolites in nature. It would be a great breakthrough to design more therapeutic itaconate derivatives to mimic the treatment of itaconate *in vivo*, but more analysis is needed at the beginning of clinical trials or the further structural based drug design to produce a better effect.

## Author Contributions

JL and JR designed the main ideas and wrote the article. YD was responsible for literature collection. DG was mainly responsible for language refinement and picture drawing. LY guided the whole process. All authors contributed to the article and approved the submitted version.

## Conflict of Interest

The authors declare that the research was conducted in the absence of any commercial or financial relationships that could be construed as a potential conflict of interest.
